# Transfer and Multi-task Learning in QSAR Modeling: Advances and Challenges

**DOI:** 10.3389/fphar.2018.00074

**Published:** 2018-02-06

**Authors:** Rodolfo S. Simões, Vinicius G. Maltarollo, Patricia R. Oliveira, Kathia M. Honorio

**Affiliations:** ^1^School of Arts, Sciences and Humanities, University of São Paulo, São Paulo, Brazil; ^2^Department of Pharmaceutical Products, Faculty of Pharmacy, Federal University of Minas Gerais, Belo Horizonte, Brazil; ^3^Center for Natural and Human Sciences, Federal University of ABC, Santo André, Brazil

**Keywords:** drug design, medicinal chemistry, QSAR, machine learning, transfer learning, multi-task learning

## Abstract

Medicinal chemistry projects involve some steps aiming to develop a new drug, such as the analysis of biological targets related to a given disease, the discovery and the development of drug candidates for these targets, performing parallel biological tests to validate the drug effectiveness and side effects. Approaches as quantitative study of activity-structure relationships (QSAR) involve the construction of predictive models that relate a set of descriptors of a chemical compound series and its biological activities with respect to one or more targets in the human body. Datasets used to perform QSAR analyses are generally characterized by a small number of samples and this makes them more complex to build accurate predictive models. In this context, transfer and multi-task learning techniques are very suitable since they take information from other QSAR models to the same biological target, reducing efforts and costs for generating new chemical compounds. Therefore, this review will present the main features of transfer and multi-task learning studies, as well as some applications and its potentiality in drug design projects.

## Introduction

The drug design process, since the discovery/identification of bioactive compounds until the approval of its clinical use by a regulatory agency, is very complex and demands time and financial support (Tufts Center for the Study of Drug Development [CSDD], 2014). There are several well-known bottlenecks in this process, such as finding out a suitable and validated molecular target, designing and/or discovering of a lead compound, pharmacokinetic and toxicity optimization, besides commercial reasons, efficacy and clinical safety (Khanna, 2012; Medina-Franco et al., 2013). In this scenario, the use of computational techniques in drug discovery is rapidly increasing.

**FIGURE 1:**
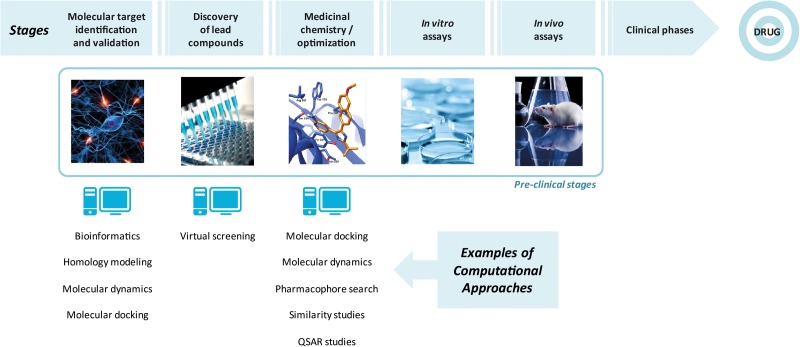
Main steps involved in drug design, highlighting the use of computational approaches.

Computer-aided drug design (CADD) techniques are broadly employed in order to reduce costs and time involved in drug design. Among the important CADD techniques, molecular docking, similarity search and QSAR studies could be highlighted. Molecular docking and virtual screening are considered structure-based drug design (SBDD) strategies since it requires 3D structure of a molecular target and consists of predicting a binding mode of molecules and its binding energy (Walters et al., 1998; Shoichet, 2004; Andricopulo et al., 2008). As the docking simulations consider both structures (ligands and targets), its calculations are more computationally expensive. Considering these aspects, similarity searches and pharmacophore modeling are alternatives to faster calculations (Brogi et al., 2009; Tresadern et al., 2009) and are defined as ligand-based drug design (LBDD) strategies since they do not require the biological target structure (Turki et al., 2017). **Figure 1** illustrates the main steps in a drug design process, including the use of computational tools.

Another LBDD strategy is known as quantitative structure-activity relationships (QSAR) and it has been widely employed in drug design, mainly aiming to predict the biological activity of a compound set against a specific target to optimize the binding affinity (Du et al., 2008; Gertrudes et al., 2012). QSAR models provide accurate predictions of measured endpoints instead of an independent ranking of biological activity. These quantitative approaches have also been used in other tasks, such as optimization of pharmacokinetics and toxicity profile (Maltarollo et al., 2015; Egeghy et al., 2016; Chemi et al., 2017) and virtual screening (Brogi et al., 2013; Melo-Filho et al., 2016; Neves et al., 2016; Zaccagnini et al., 2017).

Several important QSAR studies can be found in literature, which include the description of successful computational methods and algorithms (Sliwoski et al., 2014; Raies and Bajic, 2016), validation techniques (Gramatica and Sangion, 2016), applications (Cherkasov et al., 2014; Fang and Xiao, 2016) as well-challenges and how those have been addressed (Cronin and Schultz, 2003; Arthur, 2008; Dearden et al., 2009; Scior et al., 2009; Wang et al., 2015; Ponzoni et al., 2017).

In many recent studies, machine learning (ML) methods have been largely applied to QSAR analyses. This growth has been mainly motivated by the increasing availability of data in public repositories, the use of numerous and diverse chemical descriptors and the proposal of accurate predictive algorithms, such as support vector machines (SVMs) and artificial neural networks (Gertrudes et al., 2012; Maltarollo et al., 2013; Mitchell, 2014; Lima et al., 2016). A common application of ML techniques in CADD refers to forecast new compound class labels (e.g., “active” *versus* “inactive”) using models previously derived from available training sets (Lavecchia, 2015). In such specific situation, ML techniques are said to perform a classification learning task. In addition, other sort of learning tasks can also be considered in CADD, such as clustering and ranking (Agarwal et al., 2010).

Despite of the widespread use of ML methods in QSAR modeling, the success of such approaches critically depends on the availability of a great amount of data, which remains challenging in drug discovery. This problem is strongly related to issues involving the quality of public data sources, including imprecise representation of chemical structures and inaccurate activity information (Zhao et al., 2017). Furthermore, the nature of different experimental protocols can usually lead to data belonging to different probability distributions, which makes the use of traditional ML techniques impracticable.

The data sets available in public repositories are usually obtained from single structure-activity relationship (SAR) campaigns. This explains the several particular and linear sets of compounds that are commonly used to generate only specialized QSAR models. In most of cases, biological activities of two datasets are measured under different experimental conditions, making the link among chemical spaces difficult to be analyzed (Richter and Ecker, 2015). Furthermore, a large chemical space has activity cliffs naturally: regions in a structure/activity surface where there is a discontinuous SAR (Cruz-Monteagudo et al., 2014).

In 2014, a review on QSAR (Cherkasov et al., 2014) stated that the transferability of QSAR models is one of the challenges in QSAR modeling, since the traditional approaches have been typically designed for each target property individually. Aiming to take advantage of diverse but related available experimental data, transfer and multi-task learning techniques have been recently developed. The novelty behind these approaches is related to their ability to exploit knowledge from other related tasks to improve the learning performance, especially when a small data set is available for training.

## Transfer and Multi-Task Learning

For QSAR purposes, the data space under analysis is characterized by biological and chemical properties. In such scenario, changes in the distribution of data force the model to be rebuilt, implying to collect new training data. However, in many real-world applications, it is expensive or impossible to recollect data required to reconstruct these models. In such situations, transfer learning (or knowledge transfer) among related domains would be desirable (Pan and Yang, 2010).

**FIGURE 2:**
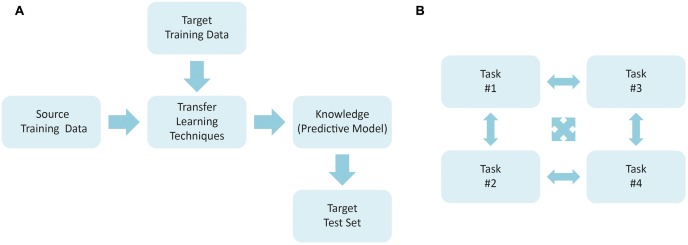
General framework used to plan a study using **(A)** transfer learning techniques and **(B)** multi-task learning.

Transfer learning can be defined as the ability of a system to recognize and apply the knowledge learned in previous (source) tasks for the solution of new (target) problems. The development of such approach was motivated by the fact that one can apply the knowledge acquired previously to solve new problems more quickly and with better solutions. The goal here relies on extracting the knowledge obtained by a model from one or more source tasks and to apply it to a target task. However, one of the premises for using transfer learning technique is that the source and the destination domains must be related. In this sense, Tan et al. (2015) suggest that such relationship can be expressed by instances (Bickel et al., 2009) or characteristics (Satpal and Sarawagi, 2007). If no direct relationship is found, the forced transfer will not work, resulting in no improvement or even degenerating the performance in the target domain (Fitzgerald and Thomaz, 2015). Multi-task learning is closely related to knowledge transfer, but they have also a clear distinction. In multi-task approaches, a number of tasks are learned simultaneously, without involving designated source and target tasks. **Figure 2** illustrates the overall schemes for transfer and multi-task learning.

**FIGURE 3:**
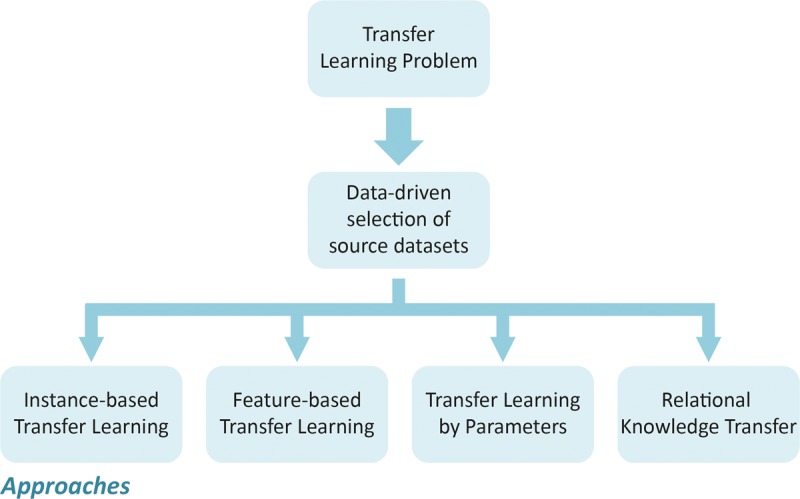
Schemes used for applying transfer learning approaches.

The methods used for transfer learning can be summarized into four categories, depending on which aspect of knowledge will be transferred, i.e., “what to transfer” (Pan and Yang, 2010). The first category refers to instance-based transfer learning, which assumes that some data from the source set can be selected for training in the target set by re-weighting. Importance sampling and instance reweighting are the two most commonly techniques used (Dai et al., 2007). The second category refers to transfer learning methods by feature representation, which focuses on encoding the structural information carried by molecules into a numerical representation that can be effectively exploited by learning processes in other related problems. In this case, the intuitive idea consists in learning a suitable representation of characteristics for use in the target set, i.e., the transfer learning is coded in the representation of the new characteristics (Raina et al., 2007). The third category refers to the transfer learning techniques by parameters (Lawrence and Platt, 2004), in which it is assumed that the source and the target tasks share some parameters or prior distributions of the hyper-parameters of the respective QSAR models. In this case, knowledge can be transferred between the tasks by discovering these shared parameters or priors. The last category consists of methods that deal with the problem of relational knowledge transfer, which refers to transfer learning in related domains (Mihalkova et al., 2007). In this condition, the knowledge can be transferred by mapping the data from the source set to the destination one. The statistical methods of relational learning are the most applied in this case (Mihalkova et al., 2007; Davis and Domingos, 2009). A scheme illustrating how the transfer learning approaches can be applied to obtain predictive models is presented in **Figure 3**.

To apply transfer learning techniques, it is assumed that two sets of related data are available and the knowledge will be transferred from the dataset with the largest volume to the set with the least amount of available data. However, this assumption in the chemical datasets is not always sufficient, requiring the opinion of an expert to define the source datasets. To overcome this limitation, Girschick et al. (2012) proposed an approach to select a source dataset in a repository containing target-related sets by following a data-driven methodology. The main idea behind such proposal is based on calculating a measure for the activity overlap between the target set and each related set available in PubChem database. As result, a ranking of all related sets according to their similarity to the target set is obtained. In order to find the similarity values, Tanimoto coefficient is calculated using the categorization of the chemical compounds (active/inactive) in each dataset. Therefore, the objective is to select the set that has the distribution of instances (compounds) closest to the distribution (number of instances categorized as active and inactive) of the target set.

One can find out many situations where transfer learning adds benefits, for example, molecules could be classified as active or inactive according to a biological data for a defined endpoint (e.g., IC_50_ values). For this classification task, it is initially necessary to collect several experimentally tested samples and, next, to train a classifier for the collected data with their respective labels. Since the probability distribution of the comments on other endpoints can be very different, a new classifier has to be trained to each dataset in order to maintain a satisfactory performance. To reduce this effort, it would be desirable to use the knowledge from a classification model that is already trained on some related endpoints to improve the classification performance of other tasks with small samples or datasets (Turki et al., 2017). **Table 1** illustrates examples of transfer learning in drug design.

**Table 1:** Examples of potential applications of transfer learning methods in drug design.

Transfer learning approach	Concept	Application in drug design
Instance-based	Uses the same ML technique for modeling but apply some changes to the parameters of the target model.	Source and target datasets have the same endpoint (e.g., same molecular target) but the training data can be colleted at different experimental conditions.
Feature representation	Based on some mathematical transformations of data.	Source and target datasets have different but related endpoints, e.g., same classes of molecular target (kinases, nuclear receptors, proteases, etc.).
Parameters	It is assumed that both datasets share some properties.	Source and target datasets have the same or related endpoints.
Relational knowledge transfer	Based on technique for mapping the data in the target domain.	The endpoints of the source and target datasets are different but the domains (the independent variable in QSAR models) are related; e.g., cellular permeability and log P.


In general, transfer learning approaches have shown to be promising for combining the knowledge previously obtained in related tasks into a single predictive model, whether for classification, regression, or grouping (Pan and Yang, 2010). In particular, researches in medicinal chemistry with focus in drug discovery have been benefited with the use of transfer learning, as can be seen in previous studies (Girschick et al., 2012; Rosenbaum et al., 2013; Saha et al., 2016). Next, applications of transfer and multi-task learning in medicinal chemistry studies will be presented.

## Some Applications of Transfer and Multi-Task Learning

Many machine learning methods are based on the assumption that similar drugs may share the same side effects, but measuring the similarity of these drugs is still a challenge. However, the use of data from various sources (similar drugs) provides important information for the analysis of side effects and should be integrated for obtaining a highly accurate prediction. Zhang et al. (2016) discussed the problem of predicting side effects caused by drugs through linear neighbor approaches and the integration of data from various sources. The authors argued that auxiliary data can bring additional and diverse information (such as drug substructures, drug targets, drug transporters, drug enzymes, drug pathways) that should be integrated to the side-effect prediction, aiming at improving its performance. Analyses on multi-label classification showed that the proposed transfer learning approaches achieved better performance than state-of-art-methods (Pauwels et al., 2011; Liu et al., 2012; Cheng et al., 2013) applied to benchmark datasets.

The task of relating chemical structure to biological activity in QSAR studies is usually based on the notion of chemical similarity to predict the molecular behavior of close compounds. So, techniques that provide similarity measures among chemical compounds are increasingly important (Floris et al., 2014). Lately, relevant solutions have been proposed, which comprise distance learning (Biehl et al., 2014) and inductive transfer (Garcke and Vanck, 2014) methods. Distance learning aims at learning an appropriate distance measure to reflect the underlying relationship between instances in the training set, while inductive transfer refers to the process of transferring knowledge learned from one task into another related task. Girschick et al. (2012) presented an adapted transfer approach, which combines distance learning and inductive transfer by learning the distances on a related task and then transferring them to the target learning task. Additionally, the authors developed a method for selecting a related task that can be used as source task for transfer learning. This technique consists in applying an activity overlap similarity measure to two datasets to find out a suitable source task. This approach was evaluated on five distinct datasets found in PubChem BioAssay (Wang et al., 2009) repository. The results showed that both proposals worked well for large and small amounts of training data.

The multi-task learning approach (Caruana, 1998) is considered to be closely related to transfer learning, since it attempts to learn multiple tasks simultaneously even when they are different. Rosenbaum et al. (2013) introduced two multi-task methods and evaluated the performance of such approaches by inferring multi-target QSAR models on a subset of human kinome. The authors assumed that the taxonomical relationship of the kinase targets should correspond to the relatedness of the QSAR problems on these targets. The multi-task techniques were compared to SVMs models independently trained for each target and an SVM model that assumed all targets to be identical. The results demonstrated that the multi-target learning can over perform baseline (pure SVM) methods if knowledge can be transferred from a target with a lot of data to a similar target with little domain knowledge.

Varnek et al. (2009) applied different inductive transfer and multi-task learning approaches to model tissue-air partition coefficients. The authors found that these techniques improved the prediction accuracy of the obtained models when compared to single task learning. Finally, this study indicated that inductive transfer learning is very suitable when single modeling is unable to generate reliable QSAR models using diverse data sets and with small amount of samples.

Brown et al. (2014) presented some challenges involved with chemogenomic data, since high-throughput assays give us a large number of information from multi-ligand and multi-target data (Pereira and Williams, 2007). So, the authors assert that computational techniques, in particular inductive transfer and explicit learning, can help to construct more robust models when compared to target-specific (classical) QSAR ones.

The study of Zhang et al. (2013) discussed the use of single- and multi-task learning to construct QSAR models for predicting the binding affinity of a compound database by estrogen receptors (ERs), which are involved with endocrine disruption by chemicals and the construction of predictive models can contribute to design safer substances. The authors concluded that multi-task learning provided better results for a small dataset (ERβ ligands) than single learning, indicating that this approach can be considered as a good tool to understand the action mechanism of endocrine disruption and to predict the ER activity of unknown compounds as endocrine disrupting chemicals.

Another interesting application of multi-task techniques was performed by Liu et al. (2011), which used multi-task learning to construct multi-target QSAR models employing three human immunodeficiency virus (HIV) inhibitor datasets together with other six subsets containing two hepatitis C virus (HCV) inhibitors. The main conclusions of this study included the fact that the integration of all databases (HIV and HCV) improved the rate of the discovery of lead HIV-HCV inhibitors, helping the design of new co-inhibitors for these important infections. Other achievement is related to the successful use (considering efficiency in convergence speed and learning accuracy) of a multi-task learning technique to construct multi-target QSAR models.

## Discussion

The main issue of transfer and multi-task learning approaches is to employ the knowledge generated (e.g., features, subset of variables, weights of equations) from available ML models and other datasets in the construction of models for related endpoints. In this sense, it is possible to use different datasets with the same biological activities but measured at different experimental conditions. Other important consequence of applying transfer and multi-task learning is the decrease on computational costs related to the faster convergence obtained by using the knowledge derived from a model previously built from a related endpoint.

From a literature review taking into to account the transfer learning applications on medicinal chemistry, one can note that there is still a great potentiality to be explored in this sense. Other emerging approaches as deep learning methods (Zhang et al., 2017), which basically use complex neural networks architectures, also have promising applications in the era of big data.

Among the main challenges on applying transfer and multi-task learning methods is that they require an artificial intelligence expert to code them since there are no chemical and/or pharmaceutical packages with a graphical user interface. Depend on the source data and on the learning method, transfer and multi-task learning could be also considered as “black-boxes,” making the interpretability of QSAR models difficult. And, finally, the transfer of knowledge could be inappropriately employed if the assumption of “equivalent” endpoints is not valid.

## Conclusion

Nowadays one can observe increasing number of applications of transfer and multi-task learning in medicinal studies. There are also current challenges in the QSAR field that comprise the integration of different datasets (even from different experiments) aiming the same or similar endpoints (Maltarollo et al., 2017) and the development of universal QSAR models using very large datasets (Alves et al., 2017). Therefore, good examples of dataset that could be benefited from transfer and multi-task learning are: (i) compounds with same endpoint measured under different experimental conditions; (ii) antimicrobial activities against genetically similar microorganisms; (iii) compounds with the same mechanism of action in homologous targets and high degree of similarity in the binding pocket; (iv) non-specific endpoints as toxicity against a cell line or permeability rates determined by different models. In this complex scenario, transfer and multi-task learning techniques can be considered powerful tools for drug design.

## Author Contributions

RS, KH, VM, and PO designed this article. All authors wrote and revised the manuscript. Also, all authors read and approved the final version of the manuscript.

## Conflict of Interest Statement

The authors declare that the research was conducted in the absence of any commercial or financial relationships that could be construed as a potential conflict of interest. The handling Editor declared a shared affiliation, though no other collaboration, with the authors.
